# The accuracy of the axial length and axial length/corneal radius ratio for myopia assessment among Chinese children

**DOI:** 10.3389/fped.2022.859944

**Published:** 2022-09-06

**Authors:** Jingfeng Mu, Dan Zeng, Jingjie Fan, Meizhou Liu, Haoxi Zhong, Xinyi Shuai, Shaochong Zhang

**Affiliations:** ^1^Shenzhen Eye Hospital, Jinan University, Shenzhen Eye Institute, Shenzhen, China; ^2^Affiliated Shenzhen Maternity & Child Healthcare Hospital, Southern Medical University, Shenzhen, China

**Keywords:** myopia, children, spherical equivalent, axial length, axial length/corneal radius ratio

## Abstract

**Objectives:**

The aim of this study was to assess the association between axial length/corneal radius ratio (AL/CR ratio), AL, and refractive status and evaluate the accuracy of AL and AL/CR ratio for myopia assessment among Chinese children.

**Methods:**

A diagnostic trial was conducted in Shenzhen Eye Hospital from June 2020 to December 2020. Cycloplegic refraction and demographic characteristic survey were carried out, and AL and CR were measured. The Pearson correlation analysis between AL, AL/CR ratio, and spherical equivalent (SE) was carried out. The sensitivity, specificity, Youden index, positive predictive value, and negative predictive value of the AL/CR ratio and AL for myopia assessment were analyzed using cycloplegic refraction as the gold standard by drawing receiver operating characteristic (ROC) curves.

**Results:**

A total of 300 children aged 8–18 years participated in this study. The Pearson correlation coefficient between AL and SE was −0.667 (*P* < 0.05) and −0.754 (*P* < 0.05) between AL/CR ratio and SE. There were significant differences in SE, AL, and AL/CR ratio among different age groups (*p* < 0.05). SE decreased by 1.185 diopter (D) for every 1 mm increase in AL and decreased by 0.667 D for every 0.1 increase in the AL/CR ratio. Taking cycloplegic refraction SE ≤ −0.50 D as the gold standard for the diagnosis of myopia, the area under the ROC curve of AL for myopia assessment was 0.836 (95% confidence interval [CI]: 0.767–0.906), with specificity, sensitivity, and Youden index of 0.833, 0.767, and 0.600, respectively. The area under the ROC curve of AL/CR ratio for myopia assessment was 0.937 (95% CI: 0.878–0.996), with specificity, sensitivity, Youden index, positive predictive value, and negative predictive value of 0.703, 0.913, 0.622, 0.956, and 0.771, respectively. The area under the ROC curve of the combination of AL/CR ratio and parental myopia for myopia assessment was 0.976 (95% CI: 0.957–0.996).

**Conclusion:**

The correlation between SE and AL/CR ratio was stronger than that between SE and AL in children. The AL/CR ratio may be an alternative indicator for myopia assessment in children, and the combination of demographic factors and AL/CR ratio can improve the accuracy of myopia assessment.

## Introduction

The increasing prevalence of myopia is a global public health problem, especially in East Asia (1). The prevalence of myopia in East Asia is as high as 50%, which is significantly higher than that in other countries (2). In recent years, the prevalence of myopia in China has increased rapidly, and the average age of myopes has decreased (3, 4). For example, the prevalence of myopia in school children in Shandong was 84.6% (5), 95.5% among university students in Shanghai (6), 36.7% in primary school children in Beijing (7), and 47.4% among primary and middle school-aged students in Guangzhou (8). It is been predicted that myopia will affect 4.7 billion people by 2050 (2).

Ocular refraction depends on axial length (AL), lens power, and corneal power (8–10). The most important influence factors of ocular refraction are AL and corneal power among children and adolescents (11, 12). There is a correlation between the AL/corneal radius ratio (AL/CR ratio) and refractive status (13). Compared with other biological parameters (such as AL, corneal curvature, and anterior chamber depth), the strongest association was found between myopia and AL/CR ratio (14). In addition, a high AL/CR ratio is a risk factor for myopia in children (15, 16). Cycloplegia refraction is the standard method to diagnose myopia in clinical settings (17). Cycloplegic refraction has problems in terms of instillation of the drop, time, mydriasis, and cycloplegia (18) and may be restricted in children (19).

The prevalence of myopia among children and adolescents is increasing in recent years. The implementation plan for comprehensive prevention and control of myopia among children and adolescents was formulated in China in 2018, and myopia assessment was conducted nationwide^1^ Therefore, measures in large-scale myopia assessment should be easily performed by technicians with limited training and with less inconvenience to participants. The easiest way for myopia assessment is to measure visual acuity and refraction without cycloplegia, which was conducted in China (20). The results of visual acuity and refraction without cycloplegia are subjective and influenced by the cooperation of participants, and the sensitivity and specificity for myopia assessment changed with the age of participants (21). The measuring results of AL and CR are precise and reliable, and the measurement of AL and CR is not invasive and is easily accepted by children.

Studies have already proved that a greater proportion of variance in spherical equivalent (SE) can be explained by AL/CR ratio, and it is superior to AL for myopia assessment among schoolchildren (15, 16), but there are few studies to evaluate the accuracy of AL and AL/CR ratio combined with demographic/behavioral factors for myopia assessment in children in China. This study aimed to assess the association between AL/CR ratio, AL, and refractive status, and evaluate the accuracy of AL, AL/CR ratio, and the combination of AL/CR ratio and demographic/behavioral factors for myopia assessment in children.

## Methods

### Subjects

Children attending the myopia clinic of Shenzhen Eye Hospital were selected as subjects by stratified random sampling based on age. A total of 300 children (151 boys and 149 girls) aged 8–18 years were enrolled from June 2020 to December 2020. The average age of the boys and girls in this study was 12.57 and 12.14 years, respectively. There was no significant difference in sex distribution among different age groups (χ^2^ = 2.426, *P* > 0.05) (Table 1). This study was conducted according to the principles of the Declaration of Helsinki, and informed consent was obtained from the parents/guardians of the participants. This study was approved by the Institutional Review Board of Shenzhen Eye Hospital (No: 20201230-06). Children with strabismus, keratopathy, cataract, glaucoma, and systemic diseases were excluded from this study.

**Table 1:** The sex characteristics of participants in this study.

**Age (years)**	**Sex**		***χ^2^***	***P*-value**
	**Boy (N)**	**Girl (N)**		
8–10	40	45	2.426	0.489
11–12	36	41		
13–14	36	35		
15–18	39	28		

All examinations were performed at Shenzhen Eye Hospital. SE was measured using an automatic refractometer (Retinomax; Nikon Inc., Ltd., Tokyo, Japan) after cycloplegia induced with five drops of 0.5% tropicamide at 5-min intervals. The AL and CR were measured in each eye using a biometer (IOL-Master 700; Carl Zeiss, Jena, Germany), which showed high reproducibility for ocular biometry (22, 23). A demographic characteristic survey was conducted in this study, including sex, age, and parental myopia.

### Data classification

High myopia, moderate myopia, mild myopia, emmetropia, and hyperopia were defined as SE ≤ −6.0 diopters (D), −6.0 D < SE ≤ −3.0 D, −3.0 D < SE ≤ −0.5 D, −0.5 D < SE≤ + 0.5 D, and SE > +0.5 D, respectively (24, 25). AL divided by CR was defined as the AL/CR ratio. Flitcroft's study (26) highlighted that the current consensus threshold value for myopia using a SE ≤ −0.50 D carried significant risks of classification bias. Spherical and cylindrical powers of emmetropic and hyperopic subjects in this study were analyzed, and all of them were emmetropic or hyperopic in both meridians.

### Statistical analysis

The Kolmogorov–Smirnov test was performed to verify the normality of SE, AL, CR, and AL/CR ratio. The refractive parameters collected in this study are normally distributed (SE: Kolmogorov-Smirnov *Z* = 1.320, *P* = 0.061; AL: Kolmogorov-Smirnov *Z* = 0.726, *P* = 0.668; CR: Kolmogorov-Smirnov *Z* = 0.624, *P* = 0.831; and AL/CR ratio: Kolmogorov-Smirnov *Z* = 0.625, *P* = 0.830). Mean and standard deviation (SD) were used to represent the concentration trend and discrete trends, respectively. The relationship between AL/CR ratio, AL, and SE was assessed using Pearson's correlation, and multiple linear regression was used to analyze the mathematical relationship between AL/CR ratio, AL, and SE. As SE, AL, and AL/CR ratio of the two eyes were highly correlated (Pearson's correlatio*n* = 0.921, *p* < 0.05), we selected the right eyes of participants to evaluate the accuracy of AL and AL/CR ratio for myopia assessment. SE, AL/CR ratio, and AL were compared between groups using a one-way analysis of variance (ANOVA). The sensitivity, specificity, Youden index, positive predictive value, and negative predictive value of AL/CR ratio and AL for myopia assessment were analyzed. The accuracy of AL/CR ratio and AL for myopia assessment was evaluated using receiver operating characteristic (ROC) curves and the area under the curve (AUC) of the ROC curves. R software version 4.1.0 (R Foundation for Statistical Computing, Vienna, Austria) was used for the analyses, and *P* < 0.05 was considered statistically significant.

## Results

### Refractive parameters according to demographic characteristics

**Figure 1:**
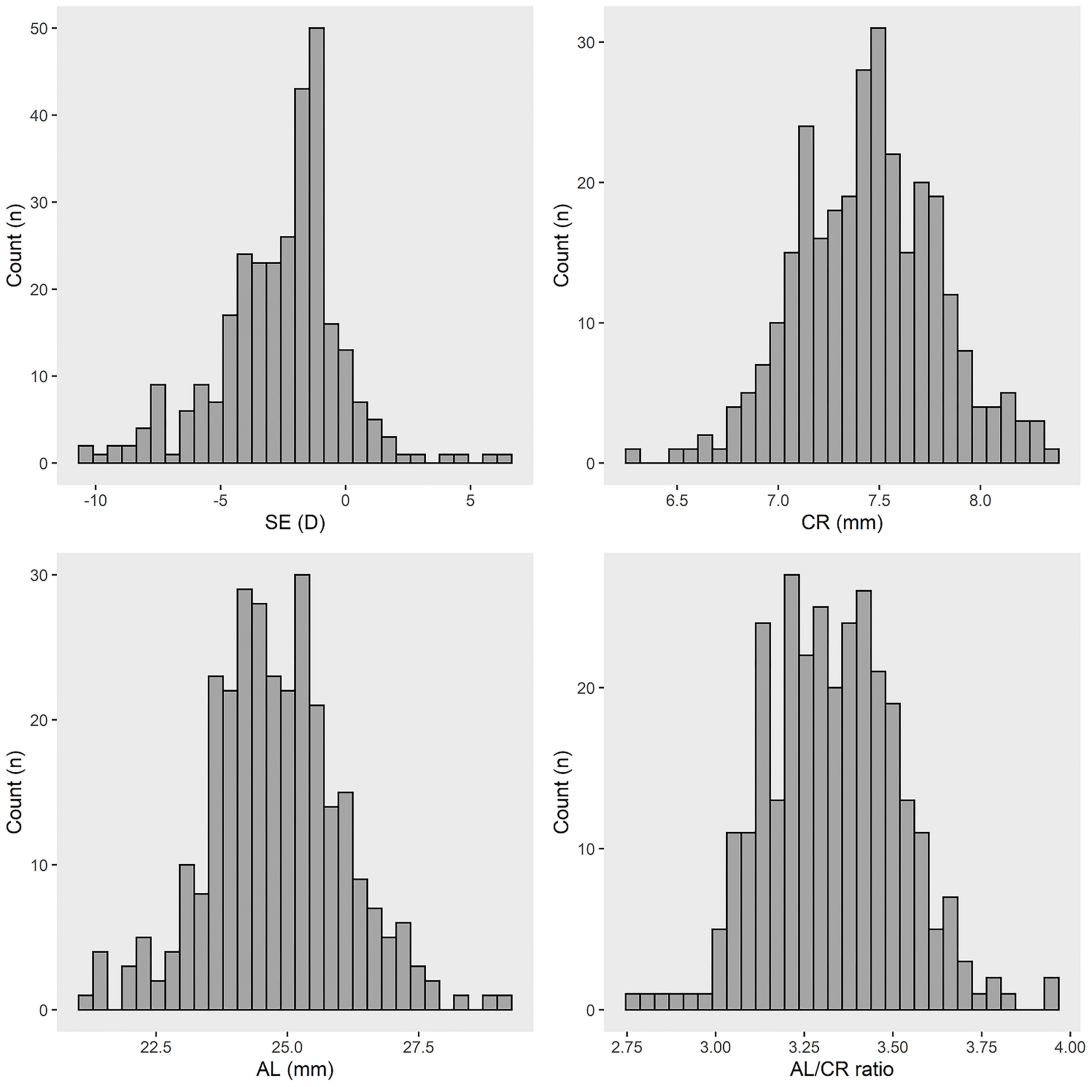
The refractive parameters of participants in this study. SE, spherical equivalent; CR, corneal radius; AL, axial length; AL/CR ratio, axial length/corneal radius ratio; D, diopter.

The histogram of refractive parameters among participants in this study is shown in Figure 1. A total of 263 (87.67%) participants suffer from myopia, and 21 (7.00%) participants suffer from hyperopia. The average SE, AL, and AL/CR ratio among participants was −2.62 ± 2.48 D, 23.78 ± 1.29 mm, 3.13 ± 0.19, respectively. SE, AL, and the AL/CR ratio were significantly different among different age groups (SE: *F* = 13.089, *P* < 0.05; AL: *F* = 16.113, *P* < 0.05; AL/CR ratio: *F* = 5.220, *P* < 0.05). AL and AL/CR ratio among 8-to-10-year-old children are 23.07 ± 0.97 mm and 3.07 ± 0.18, respectively, and increase to 24.37 ± 1.44 mm and 3.18 ± 0.18 among 15-to-18-year-old children, respectively. SE among 8-to-10-year-old children is −1.36± 1.98 D and decreases to −3.49 ± 2.50 D among 15-to-18-year-old children (Table 2).

**Table 2:** The comparison of SE, AL, and AL/CR ratio of participants among different age groups.

**Age (years)**	**SE (D)**	**AL (mm)**	**AL/CR ratio**	**The prevalence of myopia**
	**Mean ±SD**	**Mean ±SD**	**Mean ±SD**	**%**
8–10 (*n =* 85)	−1.36 ± 1.98	23.07 ± 0.97	3.07 ± 0.18	72.9
11–12 (*n =* 77)	−2.66 ± 2.24	23.89 ± 1.17	3.16 ± 0.18	88.3
13–14 (*n =* 71)	−3.27 ± 2.67	23.94 ± 1.23	3.14 ± 0.21	90.1
15–18 (*n =* 67)	−3.49 ± 2.50	24.37 ± 1.44	3.18 ± 0.18	98.5
Statistical value	*F* = 13.089	*F* = 16.113	*F* = 5.220	*χ^2^* = 22.909
*P* value	<0.001	<0.001	0.002	<0.001

SE, spherical equivalent; AL, axial length; AL/CR ratio, axial length/corneal radius ratio; D, diopter; SD, standard deviation.

### Correction analysis between SE, AL/CR ratio, and AL

**Figure 2:**
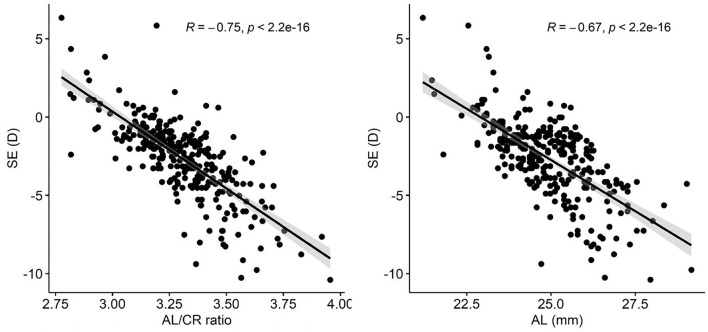
The correlation analysis between SE, AL, and AL/CR ratio. SE, spherical equivalent; AL, axial length; AL/CR ratio, axial length/corneal radius ratio; R, Pearson correlation coefficient; D, diopter.

The scatter plots between AL/CR ratio, AL, and SE are shown in Figure 2. The Pearson correlation coefficient (*r*) between SE and AL was −0.667 (*P* < 0.05) and −0.754 (*P* < 0.05) between SE and AL/CR ratio. The AL of participants with high myopia, moderate myopia, low myopia, emmetropia, and hyperopia was 25.63 ± 1.05, 24.46 ± 0.84, 23.34 ± 0.88, 22.93 ± 0.62, 21.62 ± 1.29 mm, respectively (*F* = 61.154, *P* < 0.05). The AL/CR ratio of participants with high myopia, moderate myopia, low myopia, emmetropia, and hyperopia was 3.37 ± 0.14, 3.22 ± 0.16, 3.08 ± 0.14, 2.97 ± 0.13, 2.85 ± 0.16 mm, respectively (*F* = 40.963, *P* < 0.05) (Table 3).

**Table 3:** The AL/CR ratio and AL of participants among different refractive status groups.

**Refractive status**	**AL (mm)**			**AL/CR ratio**		
	**Mean ±SD**	***F***	***P*-value**	**Mean ±SD**	***F***	***P*-value**
High myopia (*n =* 29)	25.63 ± 1.05	61.154	< 0.001	3.37 ± 0.14	40.963	<0.001
Moderate myopia (*n =* 90)	24.46 ± 0.84			3.22 ± 0.16		
Low myopia (*n =* 144)	23.34 ± 0.88			3.08 ± 0.14		
Emmetropia (*n =* 16)	22.93 ± 0.62			2.97 ± 0.13		
Hyperopia (*n =* 21)	21.62 ± 1.29			2.85 ± 0.16		

AL, axial length; AL/CR ratio, axial length/corneal radius ratio; SD, standard deviation.

**Figure 3:**
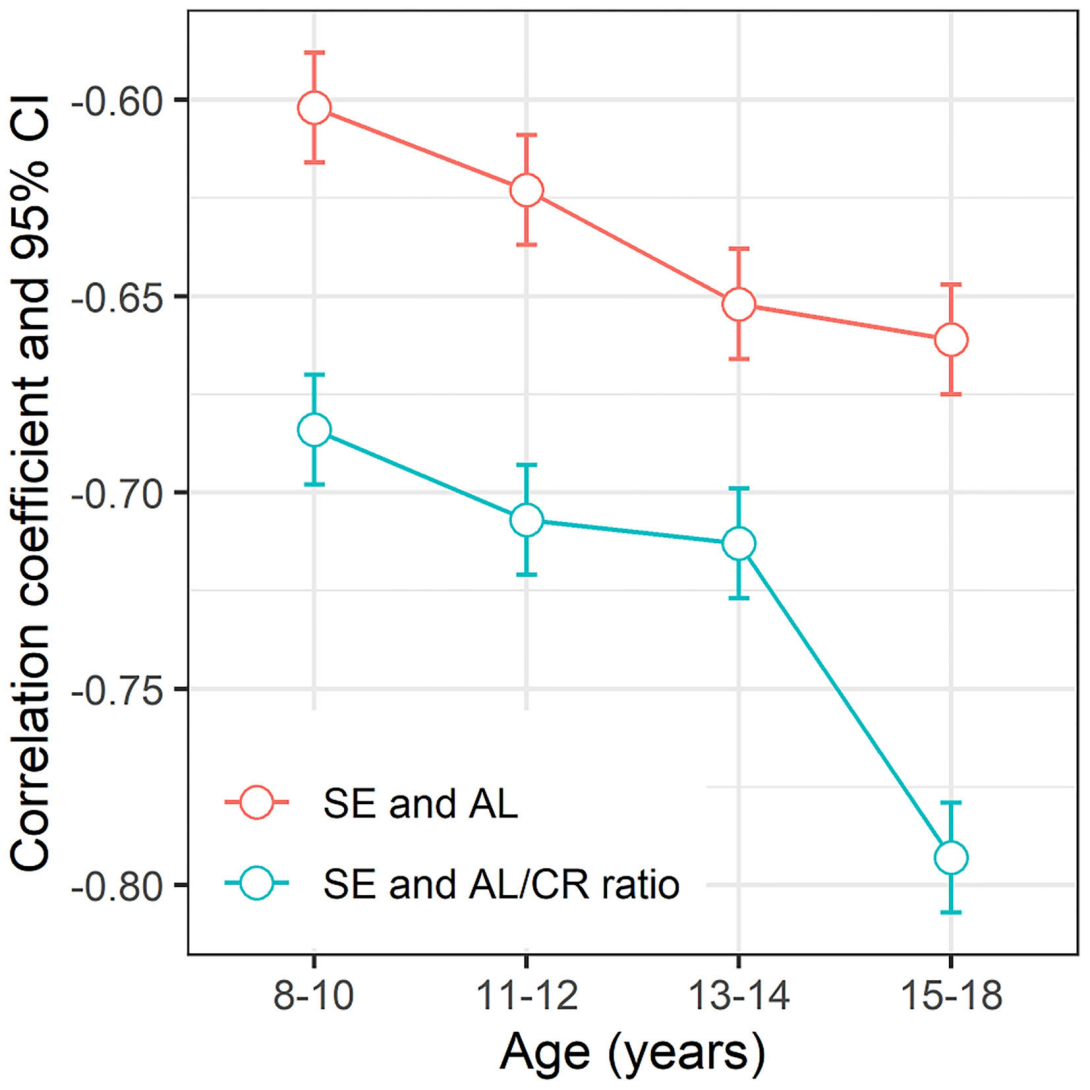
The correlation coefficient and 95% CI between SE, AL/CR ratio, and AL among different refractive status groups. SE, spherical equivalent; AL, axial length; AL/CR ratio, axial length/corneal radius ratio; 95% CI, 95% confidence interval.

The Pearson correlation coefficient between age and AL was 0.218 (*P* < 0.05), 0.394 (*P* < 0.05) between age and AL/CR ratio, and −0.358 (*P* < 0.05) between age and SE. The *r* between SE and AL/CR ratio among high myopia, moderate myopia, low myopia, emmetropia, and hyperopia groups was−0.459, −0.437, −0.420, −0.403, and −0.438, respectively (*F* = 4.280, *P* < 0.05). The *r* between AL and SE in these groups was−0.406, −0.345, −0.178, −0.112, and −0.494, respectively (*F* = 5.033, *P* < 0.05) (Figure 3).

### Regression analysis between SE, AL/CR ratio, and AL

Multiple linear regression analyses were performed in this study (Table 4). The regression equation between SE and AL was SE = −1.185^*^AL −0.133^*^sex −0.082^*^age −1.964^*^parental myopia+ 23.180. AL and parental myopia accounted for 45.2% and 8.6% of the total variance in SE, respectively (*F* = 91.24, *P* < 0.05). When other variables were fixed, SE decreased by 1.185 D for every 1 mm increase in AL. The regression equation between the AL/CR ratio and SE was SE = −6.669^*^AL/CR ratio −0.067^*^sex −0.125^*^age −2.310^*^parental myopia+ 33.418. AL/CR ratio and parental myopia accounted for 56.9 and 6.0% of the total variance in SE, respectively (*F* = 128.27, *P* < 0.05). When other variables were fixed, the SE decreased by 0.667 D for every 0.1 increase in AL/CR ratio.

**Table 4:** Multiple linear regression analysis of SE, AL, AL/CR ratio.

**Parameters**	**Regression coefficient**	**Standard error**	**Standardized coefficients**	***t***	***P-*value**	***R*-squared**
**SE and AL**						
Constant	23.180	1.760		12.998	<0.001	–
AL	−1.185	0.079	−0.516	−15.067	<0.001	0.452
Sex^#^	−0.133	0.176	−0.127	−0.756	0.450	–
Age	−0.082	0.036	−0.087	−2.315	0.021	0.015
Parental myopia^*^	−1.964	0.282	−0.270	−6.969	<0.001	0.086
**SE and AL/CR ratio**						
Constant	33.418	1.783		16.589	<0.001	–
AL/CR ratio	−6.669	0.583	−0.578	−11.440	<0.001	0.569
Age	−0.125	0.039	−0.101	−3.193	0.002	0.006
Sex^#^	−0.067	0.195	−0.013	−0.342	0.732	–
Parental myopia^*^	−2.310	0.583	−0.307	−7.469	<0.001	0.060

# Boys were used as references.

* Parents who are not myopic were used as references.

AL, axial length; AL/CR ratio, axial length/corneal radius ratio.

### Accuracy of AL/CR ratio and AL for myopia assessment

**Figure 4:**
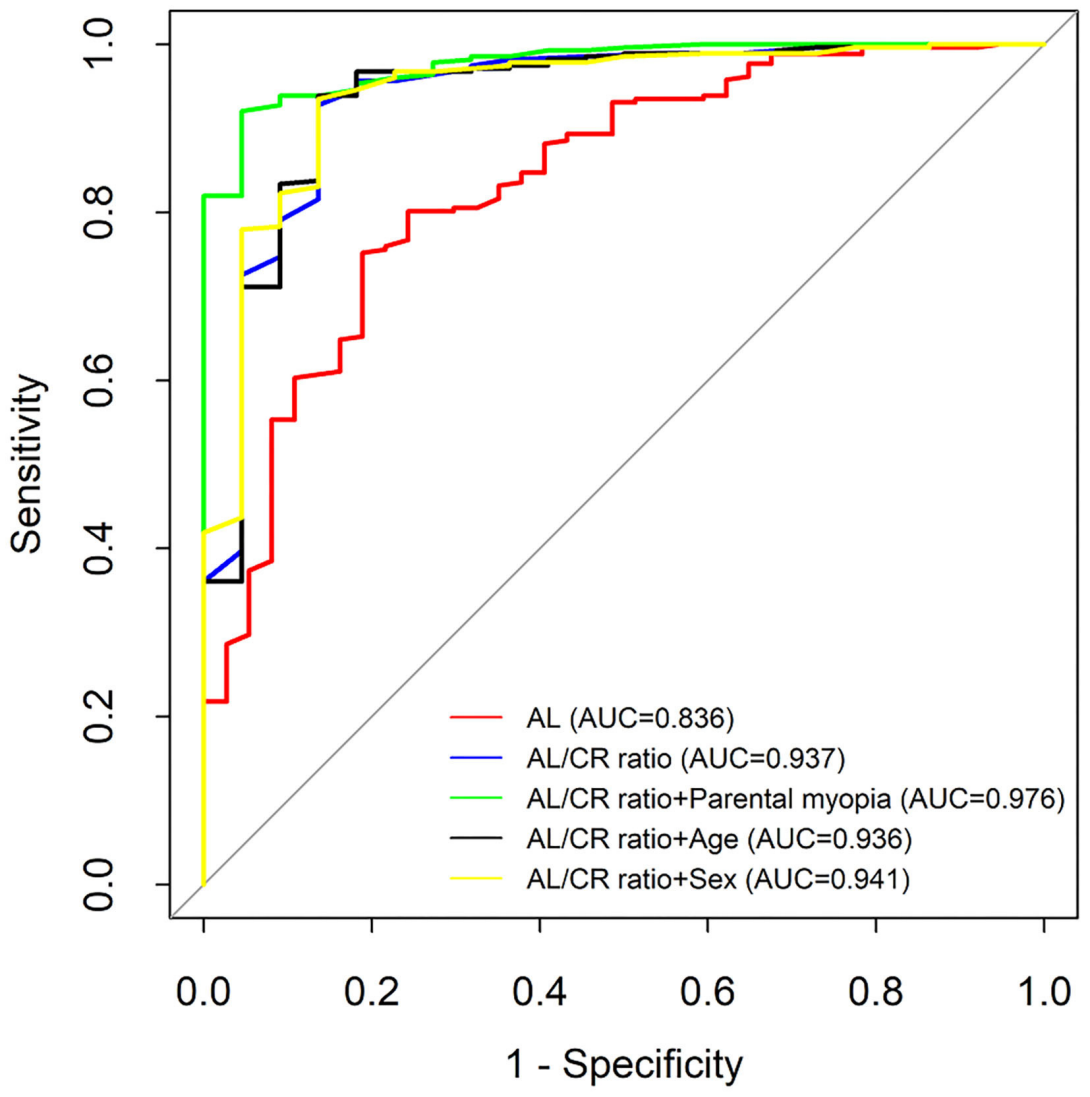
The comparison of the accuracy of AL and AL/CR ratio for myopia assessment. AL, axial length; AL/CR ratio, axial length/corneal radius ratio; AUC, area under the curve.

Taking cycloplegic refraction SE ≤ −0.50 D as the gold standard for diagnosis of myopia, the accuracy of AL/CR ratio and AL for myopia assessment were analyzed. The ROC curves were drawn using AL, AL/CR ratio, AL/CR ratio combined with parental myopia, AL/CR ratio combined with age, and AL/CR ratio combined with sex as the index for myopia assessment, and the AUC of ROC curves was 0.836 (95% confidence interval [CI]: 0.767–0.906), 0.937 (95% CI: 0.877–0.997), 0.976 (95% CI: 0.957–0.996), 0.936 (95% CI: 0.875–0.998), and 0.941 (95% CI: 0.887–0.996), respectively (Figure 4). If AL was used for myopia assessment, the optimal cutoff point of ROC curve was ≥23.63 mm, with the specificity, sensitivity, Youden index, true positive rate, and false positive rate of 0.833, 0.767, 0.600, 0.767 and 0.167, respectively. If AL/CR ratio was used for myopia assessment, the optimal cutoff point of the ROC curve was ≥3.035, with the specificity, sensitivity, Youden index, true positive rate, and false positive rate of 0.703, 0.913, 0.622, 0.913, and 0.297, respectively (Table 5).

**Table 5:** The sensitivity, specificity, and Youden index of AL/CR ratio and AL for myopia assessment.

**Criterion**	**Sensitivity**	**Specificity**	**Youden index**
**AL (mm)**			
≥23.59	0.779	0.762	0.541
≥23.60	0.775	0.810	0.585
≥23.61	0.771	0.810	0.581
≥23.63	0.767	0.833	0.600
≥23.65	0.760	0.833	0.593
≥23.66	0.752	0.833	0.585
≥23.67	0.748	0.833	0.581
**AL/CR ratio**			
≥3.005	0.958	0.574	0.532
≥3.015	0.951	0.601	0.552
≥3.025	0.935	0.655	0.590
≥3.035	0.913	0.703	0.616
≥3.045	0.890	0.709	0.599
≥3.055	0.875	0.709	0.584
≥3.065	0.867	0.709	0.576

AL, axial length; AL/CR ratio, axial length/corneal radius ratio.

**Figure 5:**
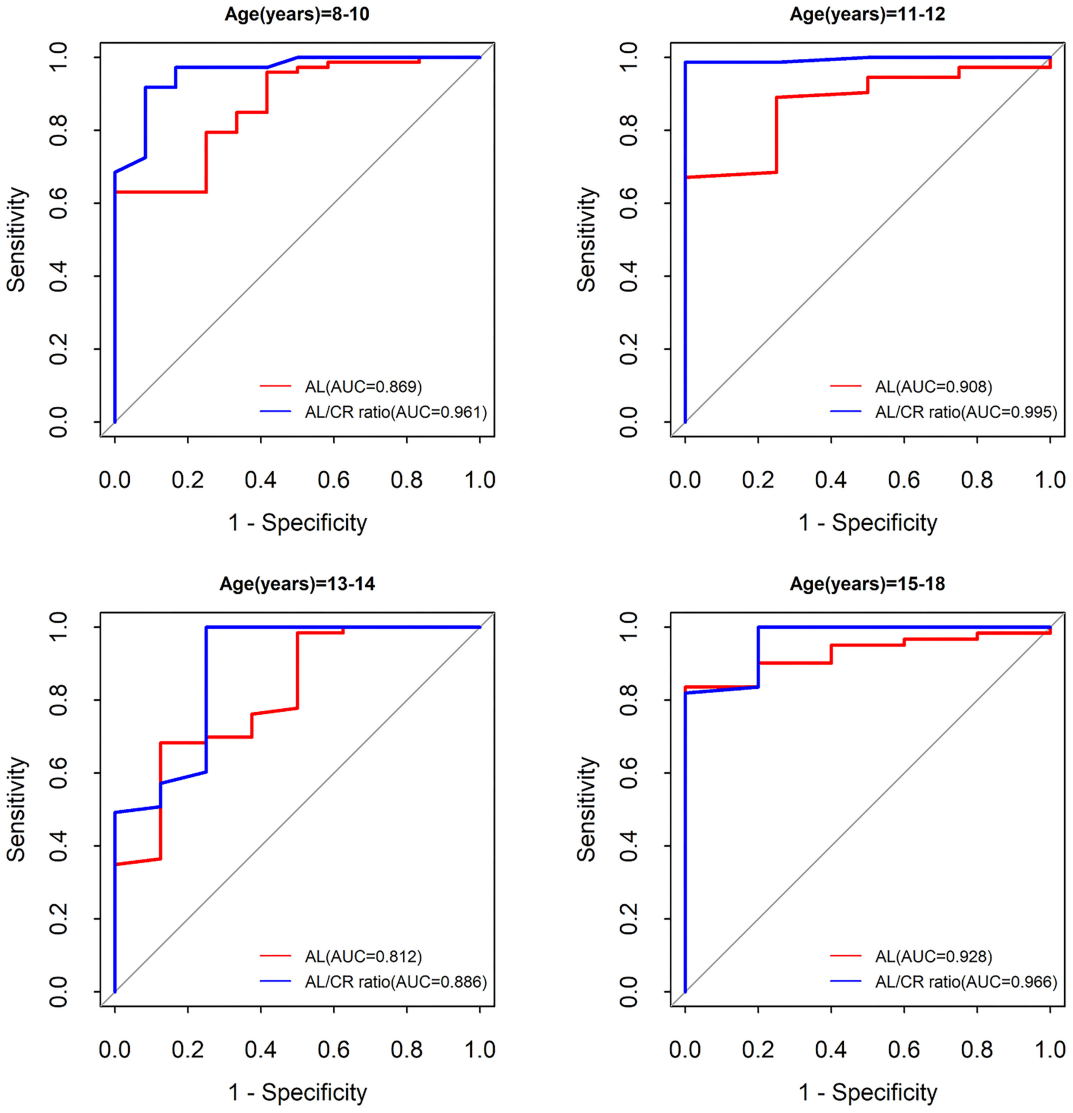
The comparison of the accuracy of AL and AL/CR ratio for myopia assessment according to age. AL, axial length; AL/CR ratio, axial length/corneal radius ratio; AUC, area under the curve.

Compared with AL, the AL/CR ratio had a higher sensitivity, Youden index, and the AUC of the ROC curve for myopia assessment. The AUC of ROC curve for AL/CR ratio was greater than that of AL (8–10-year-old children: 0.961 vs. 0.869, *P* < 0.05; 11–12-year-old children: 0.995 vs. 0.908, *P* < 0.05; 13–14-year-old children: 0.886 vs. 0.812, *P* < 0.05; and 15–18-year-old children: 0.966 vs. 0.928, *P* < 0.05) (Figure 5). Therefore, AL/CR ratio is more suitable for myopia assessment than AL. With SE ≤ −0.50 D after cycloplegia as the gold standard for the diagnosis of myopia, the positive and negative predictive values of the AL/CR ratio for myopia assessment were 0.956 and 0.771, respectively (Table 6). The AUC of the ROC curve of the combination of AL/CR ratio and parental myopia for myopia assessment was 0.976 (95% CI: 0.957–0.996), which was greater than that of the AL/CR ratio (*P* < 0.05), the combination of AL/CR ratio and sex (*P* < 0.05), and the combination of AL/CR ratio and age (*P* < 0.05) (Figure 4).

**Table 6:** SE and AL/CR ratio for myopia assessment in children.

**AL/CR ratio**	**SE (cycloplegia refraction)**		**Total**
	**≤ −0.50 D**	**>−0.50 D**	
≥3.035	240	11	251
<3.035	23	26	49
Total	263	37	300

AL/CR ratio, axial length/corneal radius ratio; SE, spherical equivalent; D, diopter.

## Discussion

In this study, we found that the correlation between SE and AL/CR ratio is stronger than that between SE and AL in children. The accuracy of the AL/CR ratio (especially the combination of AL/CR ratio and parental myopia) for myopia assessment was higher than that of AL.

Myopia is a global public health concern (27). In recent years, the prevalence of myopia in children is rising rapidly (4). The prevalence of myopia among primary and secondary school students in Shenzhen, China was 50.5% in 2020 (28). Notably, 85.0% of myopic children suffer from mild myopia in China (16), and it is very important to carry out myopia assessment and intervention for the control of myopia. It is generally believed that the main cause of myopia in school-age children is excessive axial elongation (12, 14). However, some children with shorter AL suffer from myopia, and some children with longer AL suffer from hyperopia. This is due to the fact that the refractive status is determined by the balance of the lens, AL, and CR of the eye (29).

Refractive parameters of 8–18-year-old children such as AL, SE, and CR were measured and analyzed in this study. Consistent with other studies (17), the refractive parameters collected in this study are normally distributed. AL/CR ratio and AL increased with the increase of age in this study, which is consistent with Twelker's study (30). The AL of the 8–10-year-old children (23.07 mm) in this study was larger than that in Singapore (31), Australia (25), and Shanghai (17). The AL/CR ratio in the 8–10-year-old group among participants was 3.07, which increased to 3.16 in the 11–12-year-old group, which was larger than that in Shanghai (17). The correlation between AL/CR ratio and SE in emmetropia children was higher than that in hyperopes and myopes. The possible reason is that lens get thin during the early stages of myopia, and this may impact the AL/CR ratio relationship with SE to a different extent in different age groups (32), and the changes in AL/CR ratios and AL occur very fast in the early stages of myopia (32, 33). Previous studies have confirmed that the growth velocities of AL and CR are equal and remain stable during the first and second years after birth (13). CR remains stable, and AL continues to grow after 3 years of age, which leads to an increase in the AL/CR ratio (13). Therefore, the correlation between SE and AL/CR ratio gets stronger with the increasing age. When the growth velocity of AL is slower than that of the corneal curvature, the risk of hyperopia may increase (34, 35). When the growth velocity of AL is faster than that of the corneal curvature, the risk of myopia may increase (34, 35). When the growth velocity of AL and corneal curvature are equal, it may lead to emmetropia (34, 35).

We found that AL/CR ratio and AL explain 56.9 and 45.2% of the total variance of SE, respectively. SE decreased by 1.185 D for every 1 mm increase in AL and decreased by 0.667 D for every 0.1 increase in AL/CR ratio. SE decreased by 1.07 D for every 0.1 increase in AL/CR ratio according to a study conducted in Shanghai, China (17). The AL/CR ratio might be a risk factor for the development of myopia, and children with AL/CR ratio higher than 3.0 are likely to suffer from myopia (29). As shown in this study, the area under ROC of the AL/CR ratio for myopia assessment is 0.937, and the optimal cutoff value is ≥3.035, which is consistent with other studies (17, 29, 36). The area under ROC of the AL/CR ratio for myopia assessment was greater than that of AL according to different age groups. The combination of parental myopia and AL/CR ratio may improve the accuracy of myopia assessment.

Currently, work to prevent and control myopia in China is viewed as important but remains challenging. Cycloplegic refraction is commonly used to measure refraction, but it is easily influenced by the cooperation and compliance of the examinees. Therefore, cycloplegic refraction is not feasible for large-scale myopia assessment. Early diagnosis and intervention of myopia in the early stages greatly improved the prognosis of children with myopia. Therefore, it is necessary to develop a large-scale myopia assessment method with good compliance in children.

At the same time, there were some limitations in this study. First, the AL/CR ratio has been used in refractive state categorization (14). Oversimplification in using the AL/CR ratio for myopia assessment can be misrepresentative because it depends on the type of myopia in the population. If most myopia is axial myopia, perhaps the association between SE and AL/CR ratio is high. The association between SE and AL/CR ratio may not be true for other types of myopia. Second, the study population in this study came from the myopia clinic of Shenzhen Eye Hospital, and it is questionable whether the study population is representative of the children and adolescents in Shenzhen. The prevalence of myopia among the study population was 87.67%, which was much higher than that of the children and adolescents in Shenzhen in 2020 (28). There may be selection bias in this study, and we will carry out a future study to evaluate the accuracy of AL and AL/CR ratio for myopia assessment among children in the community. Third, the biggest disadvantage of cross-sectional study is the impossibility of establishing causal relationships as they do not prove the existence of a temporal sequence between exposure to the factor and the subsequent development of the disease. A cross-sectional study is one that collects and analyzes data in a time defined as observational, and its goal is to collect data to study a population at a given point in time. Furthermore, it is important to examine the relationship between variables of interest. This study can be complemented with a future longitudinal study and analyze the changes in the AL/CR ratio over time.

In conclusion, the correlation between SE and AL/CR ratio was stronger than that between SE and AL in children. The combination of parental myopia and AL/CR ratio can explain most of the total variance in SE. The detection of the AL/CR ratio is relatively objective, easy to operate, and highly acceptable to children, and parental myopia of participants can often be collected easily. The AL/CR ratio may be a good alternative indicator for myopia assessment in children, who cannot or are unwilling to undergo optometry. The AL/CR ratio can be only used to confirm axial myopia but cannot be used to confirm other types of myopia. Therefore, the AL/CR ratio cannot replace the SE measured by cycloplegic refraction in the diagnosis of myopia.

## Data availability statement

The original contributions presented in the study are included in the article/supplementary material, further inquiries can be directed to the corresponding author.

## Ethics statement

The studies involving human participants were reviewed and approved by the Ethics Committee of Shenzhen Eye Hospital. Written informed consent to participate in this study was provided by the participants' legal guardian/next of kin.

## Author contributions

Conceptualization and methodology: JM, DZ, JF, ML, HZ, XS, and SZ. Data curation: JM, DZ, JF, HZ, and XS. Formal analysis: JM, DZ, JF, ML, and SZ. Supervision: SZ. Visualization and writing—original draft: JM. Writing—review and editing: DZ, JF, ML, HZ, XS, and SZ. All authors have approved the final version of the manuscript.

## Funding

This work was supported by Sanming Project of Medicine in Shenzhen (No. SZSM202011015) and Natural Science Foundation of Guangdong Province (No. 2021A1515011090).

## Conflict of interest

The authors declare that the research was conducted in the absence of any commercial or financial relationships that could be construed as a potential conflict of interest.

## Publisher's note

All claims expressed in this article are solely those of the authors and do not necessarily represent those of their affiliated organizations, or those of the publisher, the editors and the reviewers. Any product that may be evaluated in this article, or claim that may be made by its manufacturer, is not guaranteed or endorsed by the publisher.

## Footnotes

^1^ http://www.moe.gov.cn/srcsite/A17/moe_943/s3285/201808/t20180830_346672.html
